# Comparative Study of Various *E. coli* Strains for Biohydrogen Production Applying Response Surface Methodology

**DOI:** 10.1100/2012/819793

**Published:** 2012-04-29

**Authors:** Péter Bakonyi, Nándor Nemestóthy, Katalin Bélafi-Bakó

**Affiliations:** Research Institute on Bioengineering, Membrane Technology, and Energetics, University of Pannonia, Egyetem ut 10, 8200 Veszprém, Hungary

## Abstract

The proper strategy to establish efficient hydrogen-producing biosystems is the biochemical, physiological characterization of hydrogen-producing microbes followed by metabolic engineering in order to give extraordinary properties to the strains and, finally, bioprocess optimization to realize enhanced hydrogen fermentation capability. In present paper, it was aimed to show the utility both of strain engineering and process optimization through a comparative study of wild-type and genetically modified *E. coli* strains, where the effect of two major operational factors (substrate concentration and pH) on bioH_2_ production was investigated by experimental design and response surface methodology (RSM) was used to determine the suitable conditions in order to obtain maximum yields. The results revealed that by employing the genetically engineered *E. coli* (DJT 135) strain under optimized conditions (pH: 6.5; Formate conc.: 1.25 g/L), 0.63 mol H_2_/mol formate could be attained, which was 1.5 times higher compared to the wild-type *E. coli* (XL1-BLUE) that produced 0.42 mol H_2_/mol formate (pH: 6.4; Formate conc.: 1.3 g/L).

## 1. Introduction

Hydrogen production technologies have received remarkable attention in the recent years due to the great increase in H_2_ demand as a feedstock in various industries [1]. In addition, hydrogen is considered as a clean energy carrier expected to play an important role in future fuel cells for vehicles and so forth [2]. According to some predictions, petroleum economy may transit to hydrogen economy in the next few decades if obstacles including the lack of a reliable and sufficient supply of (bio)hydrogen could be overcome [3]. Hydrogen is an ideal and completely environmental-friendly energy source, since its combustion results only in water as product [4]. Nevertheless, the conventional hydrogen production methods are disadvantageous from ecological point of view, because mostly, they are based on the steam reforming of fossil fuels (e.g., natural gas or oil) operated at high temperature and pressure [5, 6]. In contrast, biological hydrogen production from cheap, renewable resources takes place at nearly ambient circumstances, and thus offers a promising way to replace traditional methods without emitting any pollution to the environment [6, 7]. The bioprocesses for hydrogen production can be classified into two main categories: the photosynthetic and the dark fermentation processes [8]. Nowadays, anaerobic dark fermentation is more feasible for practical application compared to light-driven hydrogen bioproduction [9]. Microbes for fermentative hydrogen production either belong to strict anaerobes or facultative anaerobes [10]. Recently, a large number of microorganisms and substrates have been used and investigated for biohydrogen formation, and *Escherichia coli* has been shown as an attractive strain for bacterial hydrogen production [11–13]. This bacteria is able to generate hydrogen using various substrates, for example, glucose, lactose, formic acid, and so forth, [13, 14]. Among these alternatives, formate seems to be suitable to ensure high bioreactor performance [14]. Furthermore, formic acid can be derived from low-cost renewable materials, such as biomass [15]. Although the bioproduction of hydrogen by dark fermentative strains is attractive [16] due to its relatively high efficiency, high stability, simpler control requirements, high volumetric productivity, and so forth, it is important to notice that these systems are generally suffered from low yields (mol H_2_/mol substrate) [17]. Therefore, the substrate conversion efficiencies have to be improved in order to make the biohydrogen fermentation industrially and economically viable [17, 18]. For this purpose, metabolic engineering and process optimization appear to be an attractive technique, as it is discussed in detail in this paper. In this research, we have dealt with a comparative study of a wild-type and a genetically engineered *E. coli* microorganisms in order to show the opportunities in biohydrogen fermentation development related to metabolic strain engineering and process optimization.

### 1.1. Opportunities Related to Metabolic Engineering and Process Optimization

One of the greatest challenge for fermentative biohydrogen production is that the H_2_ yield is usually low. In theory, the complete bioconversion of glucose—the most widely used model substrate—into hydrogen could give 12 mol H_2_/mol glucose [17]. However, there are no known existing microorganisms and metabolic pathways that could be capable to do that and practical conversion efficiencies appear to be restricted to 4 mol H_2_/mol glucose using strict anaerobic bacteria, such as *Clostridium* species. In contrast, facultative anaerobe microorganisms could give 2 mol of hydrogen per mol of glucose [17]. Nevertheless, facultative anaerobes have some advantages compared to strict anaerobes for some practical reasons; for example, they are fast growing, less sensitive to oxygen, able to recover hydrogen production activity after accidental oxygen damage by rapidly depleting O_2_ present in the nutrient broth, and so forth. Therefore, a facultative anaerobe may be considered a better microorganism than a strict anaerobe to carry out fermentative hydrogen production process [8]. Among facultative anaerobes, the members of the family of *Enterobacteriaceae* are attractive for microbial hydrogen production and have been used in numerous studies [19–21]. Using these enteric-type species, such as *E. coli* 2 mol H_2_/mol glucose or 1 mol H_2_/mol formate, would be theoretically achievable [22, 23]. However, practical yields are usually less than the 50% of the predicted maximum [24, 25]. In general, the reason for only moderate hydrogen yields could be reached using wild-type microorganisms is that the substrate conversion and utilization is evolutionary optimized for bacterial growth and not for H_2_ production [26]. Thus, genetic engineering is becoming a challenging issue but such an improvement requires the metabolic pathways involved in hydrogen fermentation to be well understood [27]. Therefore, the biochemical and physiological characterization of the microbes with high hydrogen producing potential is essential for bioH_2_ process design. The approach of metabolic strain development particularly relies on molecular biology and modern analytical methods and aims to redirect and redesign metabolic network in cells by eliminating competitive pathways, increasing substrate utilization and engineering (hydrogen producing) enzymes in order to enhance productivities and/or yields [27, 28].

Besides the individual properties of the biocatalysts, the efficiency of the biological processes, such as biohydrogen production, is significantly dependent on the applied reaction conditions, as well [29]. Hence, the economic and industrial success of a biosystem needs process optimization which basically means that the key factors affecting the system performance need to be optimized. However, the optimization study in many cases represents a remarkable cost and time factor in the bioprocess development. Nevertheless, the application of statistical experimental design procedures offers a great opportunity to overcome these issues. These methods have gained noticeable attention in recent years and are being used widely in process optimization [30–32].

## 2. Materials and Methods

### 2.1. Strain Maintance

The wild-type strain, *E. coli* (XL1-BLUE), was provided by the University of Szeged (Professor Kornel Kovacs et al.). The culture was maintained in 70% glycerol at −80°C. The bacteria was subcultured on Petri plates using agar supported LB medium (10 g/L tryptone, 5 g/L yeast extract, 10 g/L sodium-chloride, 30 g/L agar). The plates were incubated at 37°C, and after 24 hours of growth, the fresh colonies were used to prepare the inoculum. The metabolically engineered strain, *E. coli* (DJT 135) was received as a kind gift from Professor Patrick C. Hallenbeck (University of Montreal, Canada). The bacteria carries mutations in uptake hydrogenases (Δ*hyd1* and Δ*hyd2*), lactate dehydrogenase (Δ*ldhA*), and *fhlA*, coding for the regulator of formate hydrogenase lyase (FHL) synthesis [33]. These modifications result in that hydrogen is not consumed by respiratory H_2_ oxidation, glucose (carbon flux) is not diverted to lactate formation, and FHL expression level is increased due to the *fhlA* modification. The cultures were maintained on Petri plates using agar supported LB medium in which 20 *μ*g/mL chloramphenicol was added. The incubation conditions were the same as for the wild-type organism.

### 2.2. Inoculum Preparation

The seed cultures for both strains were prepared aerobically in a 50 mL flask (working volume 30 mL) containing LB medium in which 10 *μ*g/mL tetracycline (XL1-BLUE), or 20 *μ*g/mL (DJT 135) was added. The application of antibiotics was twofold. For one thing, these materials help to prevent contamination. Secondly, in the case of the metabolic engineered bacteria, the appropriate concentration of chloramphenicol is needed for selective pressure to keep the plasmid. Finally, the flasks were placed in a shaking incubator at 110 rpm agitation rate (37°C, 24 h).

### 2.3. Experimental Procedure

**Figure 1:**
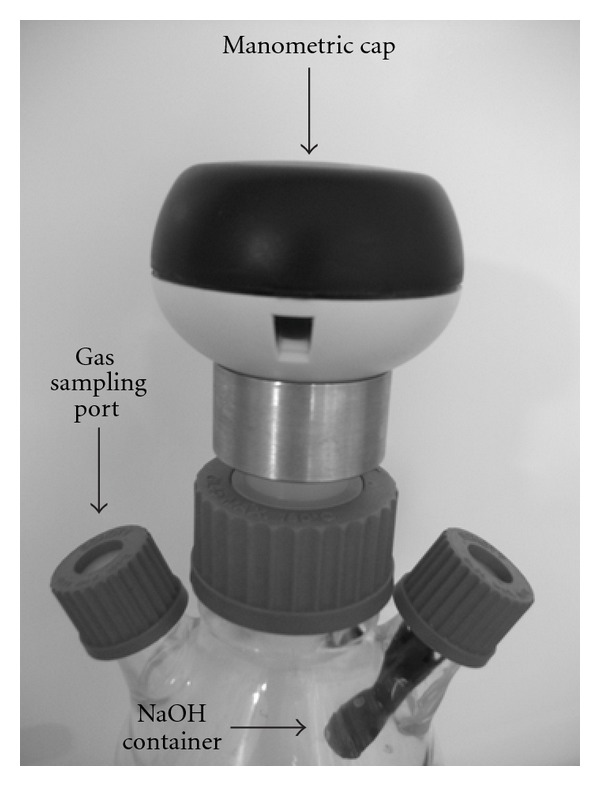
The top of the fermenter unit.

Batch experiments were carried out in WTW OXITOP 100 manometric vessels (Figure 1). The reactor was filled with phosphate buffer (various pH, Table 1) in which formate (various concentrations, Table 1), tryptone (10 g/L), yeast extract (5 g/L), and NaCl (3.33 g/L) were added. The total volume of the fermenter was 500 mL with 250 mL liquid phase containing 0.05 g dry weight cell/L. Prior to inoculation, the reactors were autoclaved for 30 min at 120°C. After cooling back to ambient temperature, the appropriate amount of antibiotic was added, and the bioreactor was purged with high purity nitrogen (>99.9%) through a sterile air filter for 10 min before start-up in order to ensure anaerobic conditions. Finally, some NaOH granulates were placed in the headspace of the fermenter, and then, the reactor was sealed by a special manometric cap which was able to record the increasing pressure of the evolving gas. The role of NaOH was to adsorb the CO_2_ formed during the fermentation, thus the pressure of bioH_2_ could be measured individually. The reliability of carbon-dioxide adsorption was checked by gas chromatography method (described elsewhere [34]) at the end of each experiments by taking samples from the reactor. The analysis of gas compositon showed that the amount of CO_2_ was only marginal, and hence, it could be neglected. The vessels were incubated at 37°C for 24 hours, and 220 rpm stirring rate was applied using a magnetic bar.

### 2.4. Process Optimization on the Basis of Response Surface Methodology

As it was stated above, optimization on the basis of the design of experiments (DOE) is considered as a reliable, time-saving, and cost-effective procedure in process development. Here, a three-level (3*^p^*, where *p* means the number of factors) full factorial design was conducted, and response surface methodology (RSM) was used to determine how the different process parameters should be set in order to achieve maximum performance. The response type of experimental designs involves generating a contour plot from the effects of process variables.

During RSM, the effects of the variables are described with a second-order polynomial approximation in a certain region

(1) $$Y=I_{0}+\sum M_{i}X_{i}+\sum M_{ii}X_{i}^{2}+\sum M_{ij}X_{i}X_{j},$$

where *Y* is the predicted response or dependent variable; *I*_0_ is model intercept; *M*_*i*_, *M*_*ii*_, and *M*_*ij*_ are linear, quadratic and interactive coefficients, respectively; *X*_*i*_ and *Xj* are independent variables. Since curvature effects are to be estimated identifying the optimum, the experimental design must have at least three levels of each independent parameters. Usually, the values assigned to these levels are equally spaced. In our study, two process variables (substrate concentration and pH) have been chosen to be investigated in order to show how the optimal operational conditions could be determined by RSM to improve hydrogen yields, as the dependent variable. The codes and the levels of the factors and the proposed experimental design for the statistical evaluation are shown in Tables 1 and 2, respectively.

A total number of 12 measurements (including 3 replications in the center point to estimate the standard deviation) were performed for both strains to predict the optimal conditions for maximal hydrogen yield, and a quadratic polynomial equation was fitted between the dependent (H_2_ yield) and independent parameters (pH, formate concentration). 

## 3. Result and Discussion

The metabolic engineering of native hydrogen-producing routes in most of the cases focuses on the enhancement of H_2_ yields on different carbon sources including the overexpression of some enzymes and redirection of carbon flux by deleting competitive metabolic pathways. Therefore, proper modification of the strains could be useful for efficient hydrogen production, but in order to realize this potential, bioprocess optimization is required, since the performance of the biosystems significantly depends on the applied reaction conditions, where pH and carbon source are obviously crucial parameters. The pH is very important, since it can influence the activity of the whole cell biocatalysts and their active metabolic pathways, and hence, it is able to affect the composition of fermentation end products. Optimum pH should be in a range that does not inhibit bacterial growth and allows high level expression of the desired fermentation route. Initial substrate (in our case formate) concentration also could have a remarkable effect on hydrogen yield and production rate. It has been reported that using pure cultures, such as *E. coli* low initial substrate, concentrations are accompanied with high yields and relatively low production rates, while higher productivities and lower yields could be achieved at higher carbon source concentrations [13, 33]. In this study, the optimal pH and substrate concentration were determined by statistical experimental design for both the wild-type and the metabolic engineered microorganisms in order to obtain improved yields, and finally, the performance of the strains was compared. Response surface methodology—which is often used in bioengineering researches—was employed to investigate the influence of two independent key variables on hydrogen yields. Therefore, 12 experimental runs were conducted at different parameter values indicated in Table 2. The pressure of hydrogen formed was measured manometrically, and the amount of H_2_ was calculated using the ideal gas equation (*n*_*H*_ = *p*_*H*_∗*V*∗*R* ^−1^∗*T* ^−1^), where *n*_*H*_ and *p*_*H*_ are the amount of substance of H_2_ and the absolute H_2_  pressure, respectively; *V* is the free gas volume; *R* is the universal gas constant; *T* is the temperature. Afterwards, the yields (mol H_2_/mol formate) could be calculated. The evaluation of the experimental design was carried out by ANOVA (analysis of variance) using Statistica 8 software and the results are presented in Table 3.

**Figure 2:**
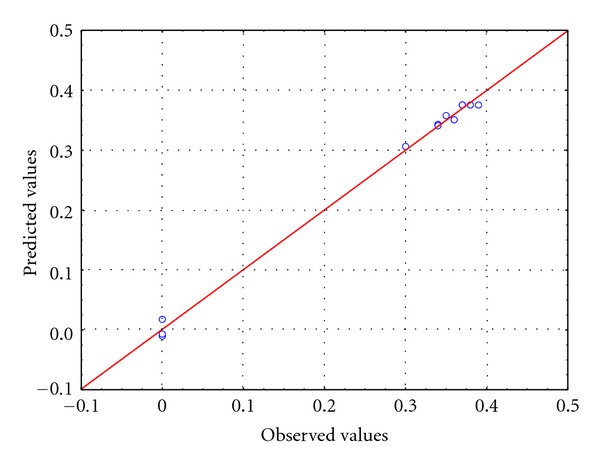
Parity plot showing the distribution of observed and predicted values of hydrogen yield for *E. coli* (XL1-BLUE).

The fitted model showed a satisfactory explanation in both cases (XL1-BLUE: *R* ^2^ = 0.993; DJT 135: *R* ^2^ = 0.995), however, not all of the effects of factors and their interaction were statistically important on hydrogen yield. According to ANOVA, parameters with significance values (*P*) lower than 0.05 (*P* < 0.05) could be considered significant, and on the basis of this assumption, formate concentration and also the pH were found to be statistically important. Nevertheless, the effect of varying the concentration of carbon source was much higher than changing the pH. ANOVA also indicated that no significant interaction occurred between the factors in the range that was under consideration. As an example, Figure 2 shows the correspondence between the observed and predicted values in the case of the *E. coli* (XL1-BLUE). The graph demonstrates that the observed and predicted values are in a good correlation which proves the reliability of the fitted model.

**Figure 3:**
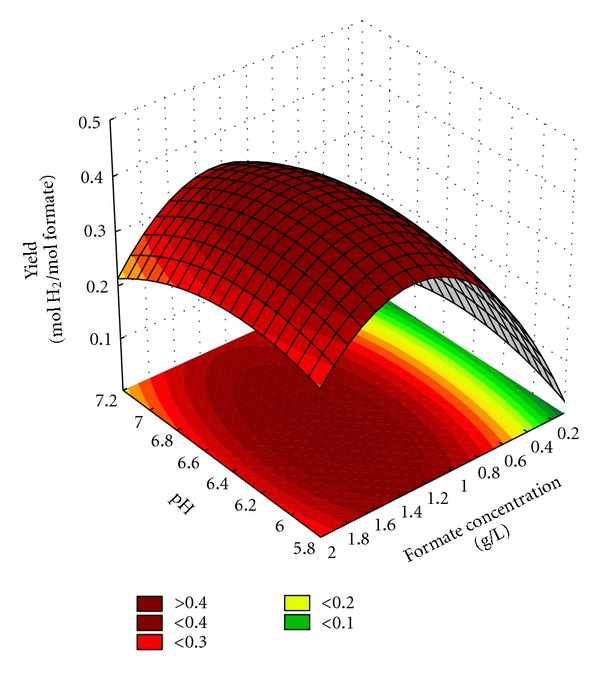
The response surface and contour plot of pH and formate concentration on hydrogen yield for *E. coli* (XL1-BLUE).

**Figure 4:**
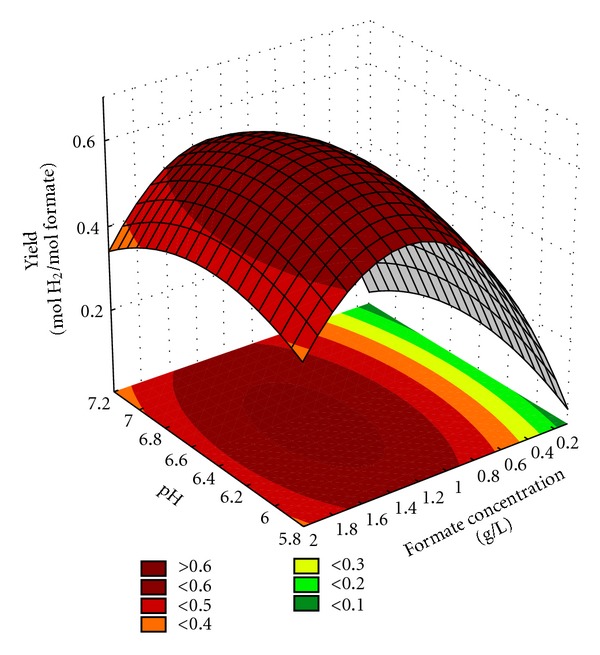
The response surface and contour plot of pH and formate concentration on hydrogen yield for *E. coli* (DJT 135).

As it can be seen in Figures 3 and 4, both response surfaces have an extremum (clear maximum peak), which means that the maximum hydrogen yield could be attained between the design coordinates.

As it can be concluded, hydrogen yield increased with increasing substrate concentration up to 1.3 g/L formate (XL1-BLUE) and 1.25 g/L formate (DJT 135), but there could be observed a decrease at higher carbon source concentrations which implied that substrate inhibition took place. Similar results were reported by Yoshida et al. [14], who have found that hydrogen formation becomes inhibited by formate concentration higher than 25 mM. We could also observe that when zero formate was added to the nutrient broth, hydrogen could not be formed probably, since proteins and amino acids are not suitable materials for hydrogen production [17], thus their decomposition does not yield any H_2_. The predicted optimal conditions for both strains are listed in Table 4, where it can be seen that the metabolic engineered *E. coli* strain provided 50% higher yield compared to the wild-type counterpart.

Moreover, the experimental data obtained here by statistical analysis clearly verify our previous findings [34], and it can be concluded that RSM is a useful tool to determine the optimum both of pH and substrate (formate) concentration in order to enhance hydrogen yields.

## 4. Conclusions

The results of present study clearly demonstrated that through specific genetic modifications, the hydrogen producing capability of different microorganisms could be significantly improved. In addition, it can be pointed out that bioprocess optimization is highly recommended to realize the benefits of metabolic engineered hydrogen producers. Therefore, the described combination of metabolic engineering and systematic experimental design should be applied in order to construct high-performance bioreactors for hydrogen production and achieve high enough biohydrogen production efficiencies that can compete with the conventional, nonrenewable methods.

## Figures and Tables

**Table 1:** The codes and levels of the variables used in the experimental design.

Variables	Coded level		
	−1	0	1
pH	6	6.5	7
Formate conc. (g/L)	0	0.9	1.8

**Table 2:** The 3^2^ experimental design matrix for evaluating data.

Run	pH	Formate conc. (g/L)	Yield (mol H_2_/mol formate)	
			XL1-BLUE	DJT 135
1	6	0	0	0
2	6	0.9	0.35	0.53
3	6	1.8	0.34	0.5
4	6.5	0	0	0
5	6.5	0.9	0.39	0.59
6	6.5	1.8	0.36	0.54
7	7	0	0	0
8	7	0.9	0.34	0.51
9	7	1.8	0.3	0.48
10	6.5	0.9	0.38	0.58
11	6.5	0.9	0.37	0.58
12	6.5	0.9	0.37	0.59

**Table 3:** The results of statistical analysis.

	*E. coli* (XL1-BLUE)			
	Regression coefficient	Standard error	*t*	*P *
Mean	0.2304	0.0039	58.8571	<0.0001
pH (L)	−0.0166	0.0102	−1.6257	0.1551
pH (Q)	0.0262	0.0076	3.4141	0.0142
Formate conc. (L)	0.3333	0.0102	32.5156	<0.0001
Formate conc. (Q)	0.1912	0.0074	24.8744	<0.0001
pH by formate conc.	−0.0200	0.0126	−1.5929	0.1622

	*E. coli* (DJT 135)			
	Regression coefficient	Standard error	*t*	*P *

Mean	0.3516	0.0057	61.2661	<0.0001
pH (L)	−0.0133	0.0151	−0.8871	0.4092
pH (Q)	0.0450	0.0113	3.9918	0.0072
Formate conc. (L)	0.5066	0.0150	33.7085	<0.0001
Formate conc. (Q)	0.2950	0.0113	26.1684	<0.0001
pH by formate conc.	−0.1000	0.0184	−0.5432	0.6066

**Table 4:** The statistically determined optimum conditions for hydrogen production.

	pH	Formate conc. (g/L)	Yield (mol H_2_/mol formate)
*E. coli* (XL1-BLUE)	6.4	1.3	0.42
*E. coli* (DJT 135)	6.5	1.25	0.63
